# Clinical features and risk factors of immune-mediated liver injury in non-small cell lung cancer patients treated with immune checkpoint inhibitors

**DOI:** 10.3389/fonc.2025.1575376

**Published:** 2025-08-13

**Authors:** Ling Yang, Chao Zhuo, Chonghuan Li, Yujing Liu, Xinyi Liu, Yibin Huang, Bingbing Wu, Jiawei Su

**Affiliations:** ^1^ Department of Infectious Disease, The First Affiliated Hospital of Guangzhou Medical University, Guangzhou, China; ^2^ Department of Clinical Medicine, Guangzhou Medical University, Guangzhou, China

**Keywords:** immune checkpoint inhibitors, immune-mediated liver injury, immune-related adverse events, clinical features, inflammatory biomarkers

## Abstract

**Purpose:**

This study investigated the clinical features, risk factors, and recurrence of immune-mediated liver injury (IMLI) in non-small cell lung cancer (NSCLC) patients treated with immune checkpoint inhibitors (ICIs).

**Methods:**

A retrospective cohort of 274 NSCLC patients receiving ICIs was analyzed. Baseline inflammatory markers, including white blood cell count (WBC), albumin levels, and prognostic nutritional index (PNI), were assessed for their association with IMLI. Risk factors were identified using logistic regression, and recurrence outcomes were analyzed.

**Results:**

IMLI incidence was 35.4%, with 15.5% of cases classified as grade 3-4. WBC ≤11.0×10^9^/L (P<0.001) and albumin ≥35 g/L (P<0.001) were independent predictors of IMLI. Among patients with IMLI, 28.9% experienced recurrence, with 17.9% classified as grade 3-4. Recurrence risk was not significantly higher than the initial onset (P=0.21).

**Conclusion:**

Low baseline inflammatory status predicts IMLI in NSCLC patients undergoing ICI therapy. Monitoring baseline inflammatory markers can guide risk stratification, and re-challenging ICIs in selected patients appears feasible without significantly increasing recurrence risk.

## Background

Immune checkpoint inhibitors (ICIs) have revolutionized the treatment of advanced non-small cell lung cancer (NSCLC), offering improved survival outcomes by enhancing T-cell mediated anti-tumor immunity (1). However, ICIs can also induce immune-related adverse events (irAEs) (2, 3), including immune-mediated liver injury (IMLI) (4–7). IMLI is a potentially serious side effect that complicates the clinical management of NSCLC patients undergoing ICI therapy (2, 8). The incidence of IMLI in NSCLC patients has been reported to vary widely, ranging from 15% to 50%, with a significant proportion of cases being severe (grade 3-4) (2, 9–12). While the pathophysiology of IMLI is still under investigation, the inflammatory status of patients prior to ICI therapy may play a role in the development of these adverse events (13, 14).

Previous studies have demonstrated the potential for inflammatory biomarkers, such as the neutrophil-to-lymphocyte ratio (NLR), platelet-to-lymphocyte ratio (PLR), and absolute eosinophil count (AEC), to predict the occurrence of various irAEs (10, 15, 16). However, the relationship between baseline inflammatory status and IMLI occurrence in NSCLC patients remains unclear. Furthermore, the impact of recurrence on the management of IMLI has not been extensively studied. This study seeks to address these gaps by evaluating the clinical features, risk factors, and recurrence of IMLI in NSCLC patients treated with ICIs, and to explore the predictive value of pre-treatment inflammatory status for IMLI.

## Methods

### Study design and population

This was a retrospective cohort study conducted at the First Affiliated Hospital of Guangzhou Medical University from November 2019 to November 2021. A total of 274 NSCLC patients who received treatment with immune checkpoint inhibitors (ICIs) at our institution were included in the analysis. All patients had histologically or cytologically confirmed NSCLC and were treated with ICIs either alone or in combination with chemotherapy.

### Data collection

Clinical and laboratory data were collected from electronic medical records. Baseline inflammatory markers, including white blood cell count (WBC), albumin level, and prognostic nutritional index (PNI), were recorded. Liver function tests, including alanine aminotransferase (ALT), aspartate aminotransferase (AST), and total bilirubin, were monitored during ICI therapy to assess the occurrence of immune-mediated liver injury.

### Outcome Measures

The primary outcome was the occurrence of IMLI, which was defined according to the Common Terminology Criteria for Adverse Events (CTCAE) version 5.0. Patients who developed liver injury during ICI treatment were classified into different grades based on the severity of liver damage. The secondary outcome was the recurrence of IMLI, defined as the reappearance of liver injury after resolution of the initial episode. Progression-free survival (PFS) and overall survival (OS) were also calculated.

### Statistical analysis

Univariate and multivariate logistic regression analyses were conducted to identify potential risk factors for IMLI development. Variables with a P-value <0.05 in the univariate analysis were included in the multivariate model. Univariate analysis of survival and calculation of hazard ratios was performed using Cox’s proportional hazards model. Multivariate analysis of survival was carried out using a backward conditional approach: variables with a p-value > 0.10 were removed in a stepwise fashion to leave only those with an independent significant relationship with survival. The Kaplan–Meier method was used to plot survival curves. Log-rank testing and Mantel–Byar time-dependent analysis were applied to assess statistically significant differences in survival. Odds ratios and Student’s t-test were used to assess associations between variables.All statistical analyses were performed using SPSS software (version [26.0 version], SPSS Inc., Chicago, IL), and a P-value <0.05 was considered statistically significant.

## Results

### Patient Characteristics

A total of 274 NSCLC patients were included in the study, with a median age of 61 ± 11 years (range:26–86 years). Among these patients, 206 (75%) were male, and 68 were female. The majority of patients were diagnosed with stage IV NSCLC (72%). 92% of patients received PD-1 inhibitor therapy, while the remaining patients received PD-L1 inhibitor or CTLA-4 inhibitor therapy (**Table 1**). Patients receiving combined targeted therapy (anti-angiogenic drugs, EGFR inhibitors) had a higher incidence of liver dysfunction, although the difference was not statistically significant (P = 0.056).

**Table 1:** Patient characteristics and the relationship between routine clinicopathological variables and incidence of immune-related adverse events in patients with NSCLC treated with ICIs.

Characteristics		Total	Without IMLI	With IMLI	P
		n=274	n=177	n=97	
		n (%)	n (%)	n (%)	
ECOG-performance status	0/1	204 (74.4%)	127 (71.8%)	77 (79.4%)	0.215
	2	70 (25.6%)	50 (28.2%)	20 (20.6%)	
Histologic subtype	Squamous	57 (20.9%)	32 (18.1%)	25 (25.8%)	0.178
	No-squamous	217 (79.1%)	145 (81.9%)	72 (74.2%)	
Clinical stage	III	48 (17.7%)	29 (16.4%)	19 (19.6%)	0.616
	IV	226 (82.3%)	148 (83.6%)	78 (80.4%)	
PD-L1 expression	positive	141 (51.6%)	92 (52.0%)	49 (50.5%)	0.930
	negative	67 (24.4%)	42 (23.7%)	25 (25.8%)	
	NA	66 (24%)	43 (24.3%)	23 (23.7%)	
KRAS status	Wildtype	127 (46.4%)	78 (44.1%)	49 (50.5%)	0.369
	Mutant	147 (53.6%)	99 (55.9%)	48 (49.5%)	
Liver metastasis	No	248 (90.6%)	159 (89.8%)	91 (93.8%)	0.244
	Yes	26 (9.4%)	20 (11.3%)	6 (6.2%)	
Combined with anti-angiogenic drugs	yes	120 (43.7%)	70 (39.5%)	50 (51.5%)	0.056
	no	154 (56.3%)	107 (60.5%)	47 (48.5%)	

### Risk factors for IMLI development

The incidence of IMLI was 35.4% (97/274), with 15.5% (15/97) classified as grade 3–4 liver injury. The median time to onset of IMLI was 85 ± 82 days (range: 15–720 days).

Patients with IMLI exhibited varying degrees of immune-related damage to other organs, including hyperthyroidism (7.1%), proteinuria (12.7%), elevated creatinine (23.8%), elevated cardiac enzymes (7.1%), and skin toxicity (0.8%). The prevalence of fatty liver in IMLI patients was 20%. Methylprednisolone treatment was administered to 47 patients (48.5%).

Univariate analysis identified several baseline inflammatory markers significantly associated with IMLI occurrence. White blood cell count ≤11.0×10^9^/L (P<0.001), albumin ≥35 g/L (P<0.001), and PNI ≥45 (P<0.05) were found to be significant predictors of IMLI development. Multivariate logistic regression analysis revealed that WBC ≤11.0×10^9^/L (P<0.001) and albumin ≥35 g/L (P<0.001) were independent risk factors for the development of IMLI (**Table 2**).

**Table 2:** Risk factors for immune related liver injury.

Characteristics	Index	With IMLI	Without IML	Univariate analysis	Mutivariate analysis
		n = 97	n = 177	OR (95% CI)	OR (95% CI)
		n (%)	n (%)	p	p
Age	≥60 years	50 (51.5%)	112 (63.3%)	0.617 (0.374-1.20)	
	<60 years	47 (48.5%)	65 (36.7%)	0.060	
Gender	male	64 (66.0%)	142 (80.2%)	0.478 (0.273-0.837)	0.461 (0.228-0.322)
	female	33 (34.0%)	35 (19.8%)	0.010	0.031
White cell count	≤11.0 ×109/L	88 (91.8%)	121 (68.4%)	5.149 (2.337-11.342)	3.183 (1.218-8.317)
	>11.0 ×109/L	9 (8.2%)	56 (31.6%)	<0.001	0.018
Neutrophil count	≤7.5 × 109/L	88 (90.7%)	155 (87.6%)	1.388 (0.612-3.146)	
	>7.5 × 109/L	9 (9.3%)	22 (12.4%)	0.433	
Albumin	≥35 g/L	77 (79.4%)	47 (26.6%)	10.649 (5.877-19.294)	12.660 (1.695-94.587)
	<35 g/L	20 (20.6%)	130 (73.4%)	<0.001	0.013
NLR	<5	96 (99%)	176 (99.4%)	0.545 (0.034-8.818)	
	≥5	1 (1%)	1 (0.6%)	0.669	
PLR	≤180	27 (27.8%)	62 (35%)	0.715 (0.417-1.229)	
	>180	70 (72.2%)	115 (65%)	0.225	
PNI	≥45	83 (85.6%)	118 (66.7%)	2.964 (1.552-5.660)	
	<45	14 (14.4%)	59 (33.3%)	<0.001	
SIPS	0	75 (77.3%)	46 (26%)	9.708 (5.425-17.374)	
	1/2	22 (22.7%)	131 (74%)	<0.001	

### Incidence of IMLI

The overall incidence of IMLI in this cohort was 35.4% (n=97). Among these, 84.5%(82/97) had grade 1–2 IMLI, and 15.5%(15/97) experienced grade 3–4 IMLI. Comparison between patients with grade 1–2 and grade 3–4 liver injury revealed no significant differences in sex, age, time to liver injury onset, or recurrence of liver dysfunction. However, among inflammation-related biomarkers, white blood cell (WBC) count (P = 0.003) and PLR (P = 0.017) were significantly higher in the grade 3–4 liver injury group. In contrast, neutrophil count, NLR, PNI, and SIPS (score 0) showed no significant differences between the two groups (**Table 3**).

**Table 3:** Comparison between patients with grade 1–2 and grade 3–4 liver injury.

Characteristics	With IMLI	Grade 1, 2	Grade 3, 4	P value
	N = 97	n = 82	n = 15	
Age	56 ± 13	60 ± 10	55 ± 12	0.111
Date of occurrence	85 ± 82	88 ± 88	68 ± 44	0.402
Gender (Male, %)	64/97	57/82	7/15	0.086
WBC	9.1 ± 1.5	9.3 ± 1.4	8.1 ± 1.6	0.003
NEU	5.5 ± 1.2	6.3 ± 1.1	5.8 ± 1.4	0.081
NLR	2.55 ± 0.58	4.7 ± 2.0	4.7 ± 1.1	0.931
PLR	226.8 ± 47.1	222.0 ± 44.9	253.2 ± 51.4	0.017
ALB	37.5 ± 2.0	36.9 ± 2.4	37.0 ± 2.1	0.892
PNI	47.9 ± 3.07	44.3 ± 3.3	43.5 ± 2.4	0.345
SIPS (Score 0, %)	75/97 (77.3%)	63/82	12/15	1.000
Recurrence of liver injury	28/97	24/82	4/18	0.838

### Recurrence of IMLI

Among the 97 patients with IMLI, 28.9% (n=28) experienced a recurrence of liver injury, with 17.9% (n=5) of these recurrences classified as grade 3-4. The recurrence rate of IMLI was not significantly higher than the initial onset of liver injury (P=0.21). The inflammatory status at the time of recurrence was similar to that at the time of initial injury (**Table 4**), indicating that the immune state had returned to baseline following treatment resolution.

**Table 4:** Comparison of the inflammatory index between at the time of baseline and recurrence.

Characteristic	Baseline	Recurrence	P value
WBC	9.1 ± 1.4	9.1 ± 1.0	0.970
NEU	6.3 ± 1.1	6.1 ± 0.6	0.542
NLR	5.1 ± 2.1	4.8 ± 1.8	0.587
PLR	227.9 ± 46.0	228.7 ± 94.4	0.597
ALB	36.4 ± 2.0	36.5 ± 1.4	0.815
PNI	43.3 ± 2.6	43.5 ± 2.9	0.754
SIPS	22/28	22/28	0.537

### Survival analysis

**Figure 1:**
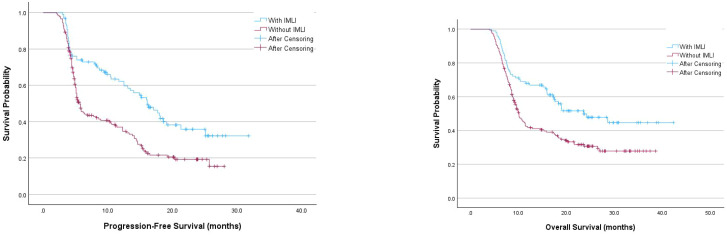
Kaplan-Meier survival curves examining the relationship between the occurrence of immune-related IMLI and progression-free survival or overall survival in patients.

The median follow-up time was 15.1 months. Patients with irAEs had a significantly longer median PFS (16.1 months vs. 5.7 months, P<0.001) and OS (23.6 months vs. 10.1 months, P<0.001) compared to those without irAEs. However, there were no significant differences in PFS and OS between patients with mild and severe AEs (P=0.560 and P=0.539, respectively) (**Figure 1**).

## Discussion

This study analyzed a cohort of 274 NSCLC patients treated with immune checkpoint inhibitors (ICIs) to investigate the clinical characteristics, risk factors, and recurrence patterns of immune-mediated liver injury (IMLI). The overall incidence of IMLI was 35.4%, with 15.5% of cases classified as grade 3–4 liver injury. Our findings highlight the significant role of baseline inflammatory status in predicting IMLI occurrence, as patients who developed IMLI exhibited a markedly lower inflammatory status prior to treatment compared to those without IMLI. Specifically, univariate analysis identified several baseline markers, including white blood cell count (WBC ≤11.0×10^9^/L), albumin levels (≥35 g/L), and the prognostic nutritional index (PNI ≥45), as significantly associated with IMLI. Multivariate analysis further confirmed that lower WBC (P = 0.018) and higher albumin levels (P = 0.013) were independent predictors of IMLI. These findings underscore the importance of baseline systemic inflammation as a key determinant of IMLI risk during ICI therapy.

Beyond traditional inflammatory markers, we also evaluated systemic inflammation-related biomarkers, including the neutrophil-to-lymphocyte ratio (NLR), platelet-to-lymphocyte ratio (PLR), and systemic inflammation prognostic score (SIPS). While NLR and SIPS did not differ significantly between patients with and without IMLI, PLR was significantly elevated in patients with severe (grade 3–4) liver injury compared to those with mild to moderate (grade 1–2) liver injury (P = 0.017). Additionally, WBC count was significantly higher in severe cases (P = 0.003). These findings suggest that while a lower baseline inflammatory status predisposes patients to IMLI, more severe cases may be characterized by an exaggerated immune response once liver injury is initiated. This observation aligns with previous reports suggesting that excessive immune activation contributes to severe immune-related adverse events (irAEs) (17).

The mechanistic basis for this association likely involves the interplay between baseline immune surveillance and the heightened immune activation induced by ICIs (18). By inhibiting the PD-1/PD-L1 and CTLA-4 pathways, ICIs enhance T-cell-mediated anti-tumor immunity but can also lead to immune dysregulation, resulting in irAEs such as IMLI (5, 6, 17). Patients with lower baseline inflammatory markers, including reduced WBC, albumin, and PNI, may have weaker baseline immune surveillance, making them more susceptible to dysregulated immune responses following ICI initiation. This hypothesis aligns with prior studies linking inflammatory biomarkers such as NLR and PLR to irAE development (19–21). Our findings extend this evidence by highlighting the predictive value of these markers specifically in IMLI. Furthermore, the association between elevated PLR and severe IMLI suggests that immune hyperactivation may be more pronounced in cases of severe liver injury. In this study, the inflammatory markers selected were white blood cells, neutrophils, and albumin. While previous research in NSCLC has identified lower baseline levels of NLR, PLR, CRP, CXCL9, CXCL10, CXCL11, and CXCL19 as associated with irAEs (2), some inflammatory markers, such as C-reactive protein (CRP) and IL-6, were not routinely measured in this cohort, potentially introducing selection bias. Future studies should incorporate a broader range of inflammatory markers to provide more comprehensive insights.

Another key finding of our study is the recurrence pattern of IMLI. Among the 97 patients who developed IMLI, 28.9% experienced recurrence, with 17.9% of these cases classified as grade 3–4. Patients who experienced recurrence were slightly younger than those who did not (mean age: 59.3 vs. 58.9 years, P = 0.047), though the difference was marginal. Notably, among patients who initially presented with grade 3–4 liver injury, 26.7% experienced recurrence, and 75% of these cases progressed to severe (grade 3–4) liver injury upon recurrence. These findings indicate that while the overall recurrence risk is moderate, patients with a history of severe IMLI may be at a higher risk of developing severe recurrence. However, inflammatory markers during recurrence were comparable to those observed during initial IMLI onset, suggesting that immune recovery after initial treatment was sufficient to restore the baseline state. Additionally, the recurrence rate was not significantly higher than the initial onset rate (P = 0.21), implying that ICI re-treatment does not substantially increase the likelihood of recurrence. These observations align with previous studies demonstrating that recurrent irAEs, while not uncommon, are often manageable and do not necessarily compromise treatment efficacy (8, 22). For instance, studies in melanoma patients have reported a low likelihood of irAE recurrence following ICI re-challenge, with minimal impact on treatment response or survival outcomes (23, 24).

From a clinical perspective, our findings have important implications for the management of IMLI and ICI re-challenge strategies. While irAEs remain a challenge in immunotherapy, our data suggest that ICI re-treatment may be feasible in selected patients with prior IMLI. This is particularly relevant for patients with recurrent IMLI, who demonstrated similar immune tolerance and recurrence risk upon re-exposure to ICIs. Moreover, the observed median time to IMLI onset (~85 days) underscores the need for close monitoring during the first three months of therapy (4, 10), particularly in high-risk patients with low baseline inflammatory status. Early identification and prompt management of IMLI during this period could help mitigate disease severity and optimize treatment outcomes. Given that nearly half (48.5%) of IMLI patients in our cohort required corticosteroid therapy (e.g., methylprednisolone), early intervention may also reduce the need for prolonged immunosuppressive treatment, thereby minimizing treatment delays and optimizing the therapeutic window of ICIs.

Current guidelines from ASCO (25), ESMO (26), and Chinese Society of Clinical Oncology (CSCO) (27) diagnosis and treatment guidelines for colorectal cancer 2018 (English version) generally recommend permanent ICI discontinuation in patients experiencing grade 3–4 irAEs. However, emerging evidence suggests that ICI re-challenge may be a viable option in carefully selected cases. Our study contributes to this growing body of literature by demonstrating that IMLI recurrence does not significantly increase following ICI re-treatment. Nonetheless, clinicians should weigh the risks and benefits on a case-by-case basis, considering factors such as the patient’s response to initial ICI therapy, baseline inflammatory status, and the severity of prior irAEs. The apparent immune recovery observed in recurrent IMLI cases suggests a potential window for safe ICI re-challenge in appropriately monitored patients. Notably, among inflammation-related biomarkers, neither NLR nor SIPS was associated with IMLI recurrence, indicating that these markers may be more relevant for predicting initial onset rather than recurrence risk.

Finally, the IMLI incidence reported in this study (35.4%) aligns with prior estimates ranging from 15% to 50%. The variability in reported incidence rates likely reflects differences in study populations, treatment regimens, and diagnostic criteria. For instance, our cohort had a high proportion of patients receiving PD-1 inhibitors, which may have influenced the observed IMLI incidence. Notably, the incidence of grade 3–4 IMLI in our study (15.5%) was higher than some prior reports, potentially due to variations in treatment combinations, such as the inclusion of ipilimumab in dual-therapy regimens. Additionally, the relatively high prevalence of fatty liver (20%) in our cohort may have contributed to an increased susceptibility to liver injury, as underlying hepatic conditions have been implicated as potential risk factors for ICI-related hepatotoxicity.

This study provides novel insights into the risk factors, recurrence patterns, and clinical management of IMLI in NSCLC patients undergoing ICI therapy. Low baseline inflammatory status, as indicated by markers such as WBC, albumin, and PNI, is a significant predictor of IMLI, highlighting the potential for these markers to be used in risk stratification and early intervention (9, 19). Additionally, IMLI recurrence does not appear to be significantly increased with ICI re-treatment, suggesting that ICI re-challenge may be feasible in selected patients. However, patients who initially present with severe IMLI should be closely monitored, as they may be at a higher risk of severe recurrence. Future research should focus on prospective validation of these findings and further exploration of the underlying immunopathology of IMLI. By integrating predictive biomarkers with individualized treatment strategies, clinicians can optimize ICI therapy while minimizing the risk of adverse events, ultimately improving outcomes for patients with NSCLC.

This study, as a retrospective analysis, has several methodological limitations. Firstly, the data collection for inflammatory markers was incomplete, resulting in a limited number of inflammatory parameters being included in the analysis. Secondly, for patients who experienced poor efficacy following IMLI, the absence of data prevented us from comparing PFS and OS between the retreatment and non-retreatment groups. Moreover, the study did not systematically analyze the potential impact of combined chemotherapy regimens on liver dysfunction. Lastly, the evaluation of ICI adverse reactions was incomplete; data on toxicity in organs other than the liver were notably absent, which may have introduced statistical bias. Future studies should aim to conduct prospective, multicenter clinical trials that focus on: 1) the mechanisms underlying the recurrence of ICI-related adverse reactions; and 2) improving long-term survival outcomes (PFS and OS) through standardized monitoring and management of adverse reactions.

## Data Availability

The raw data supporting the conclusions of this article will be made available by the authors, without undue reservation.
